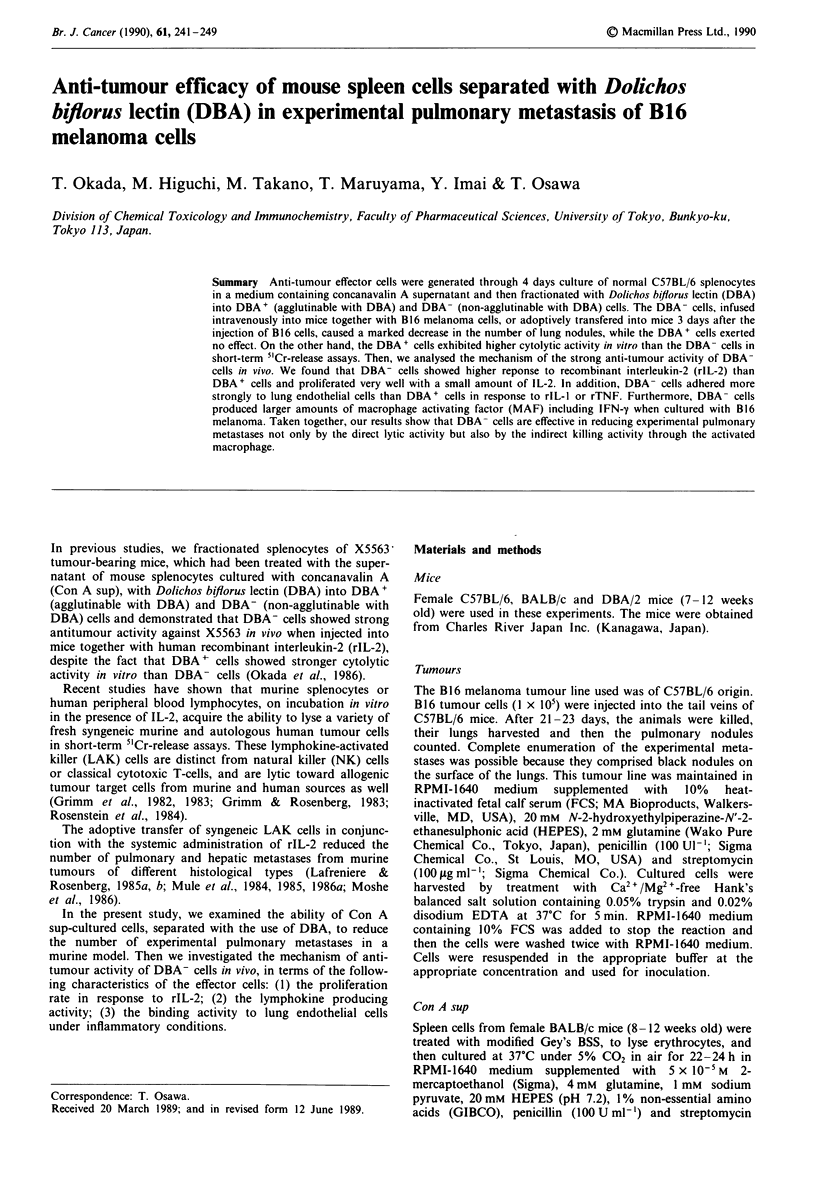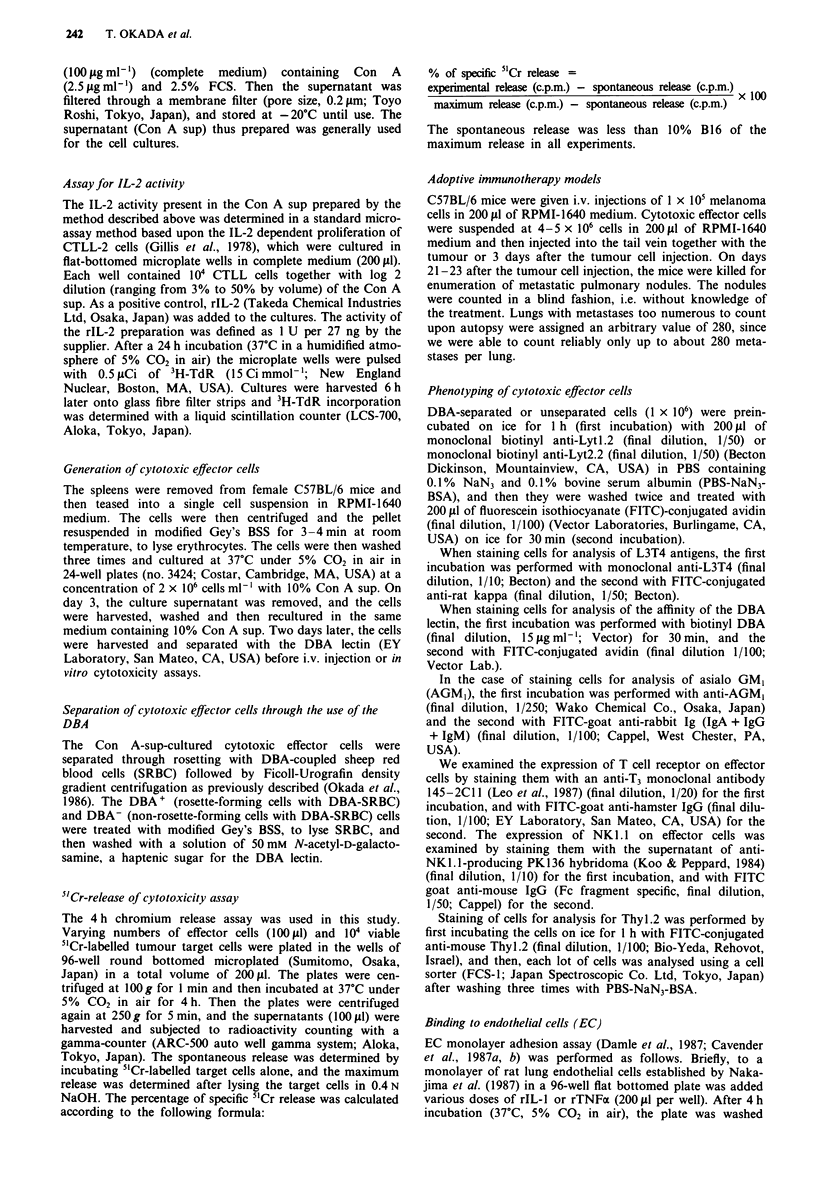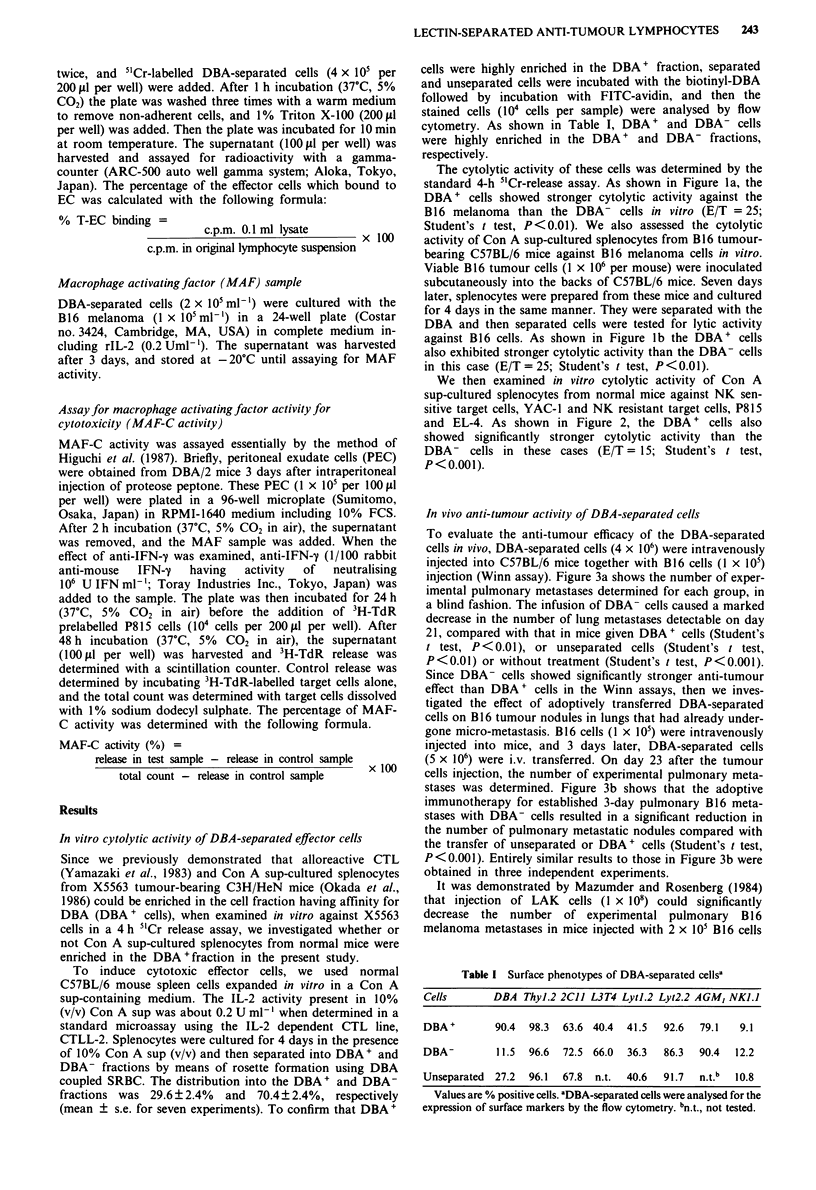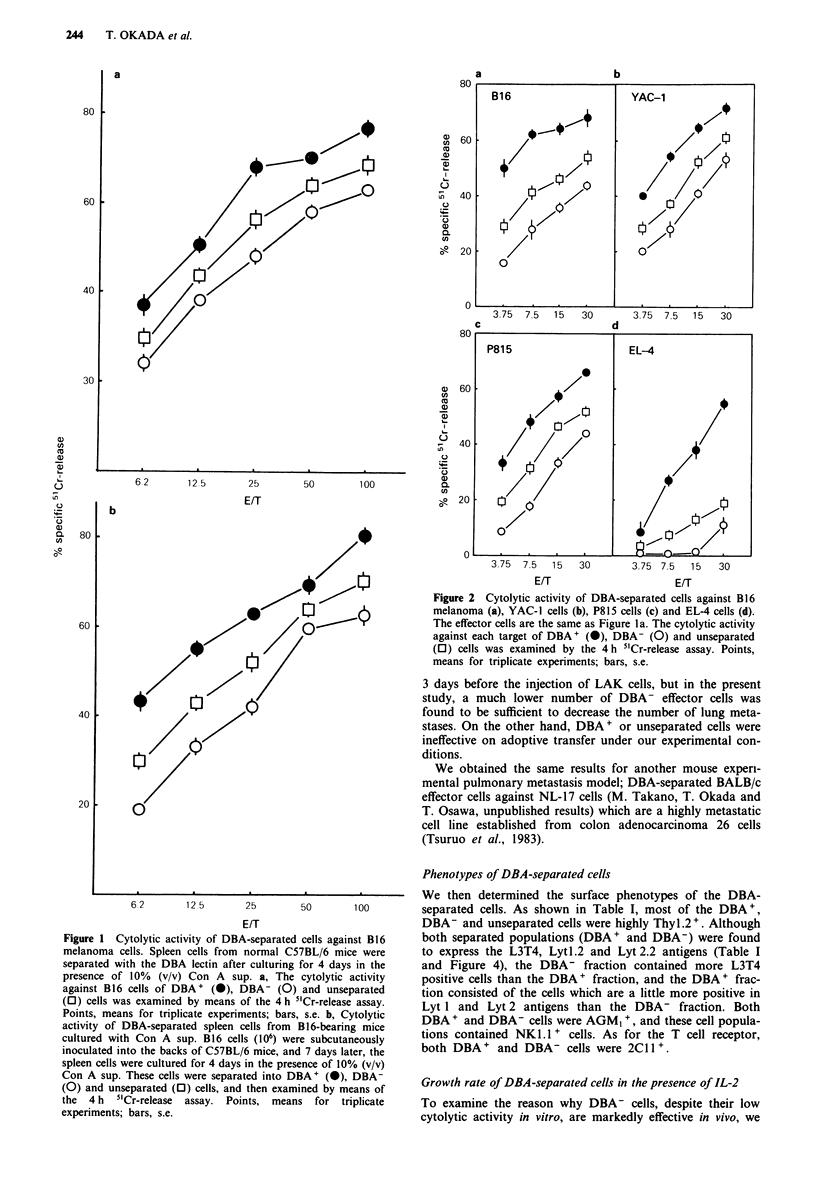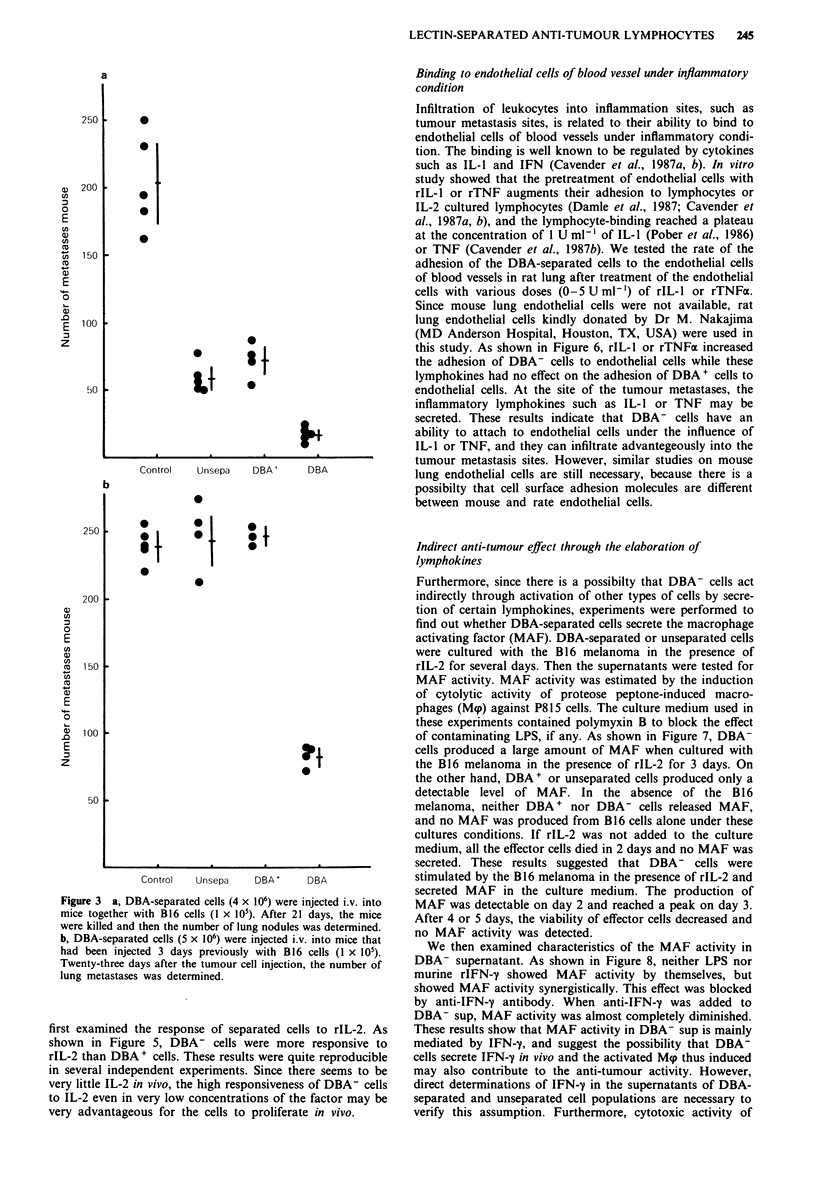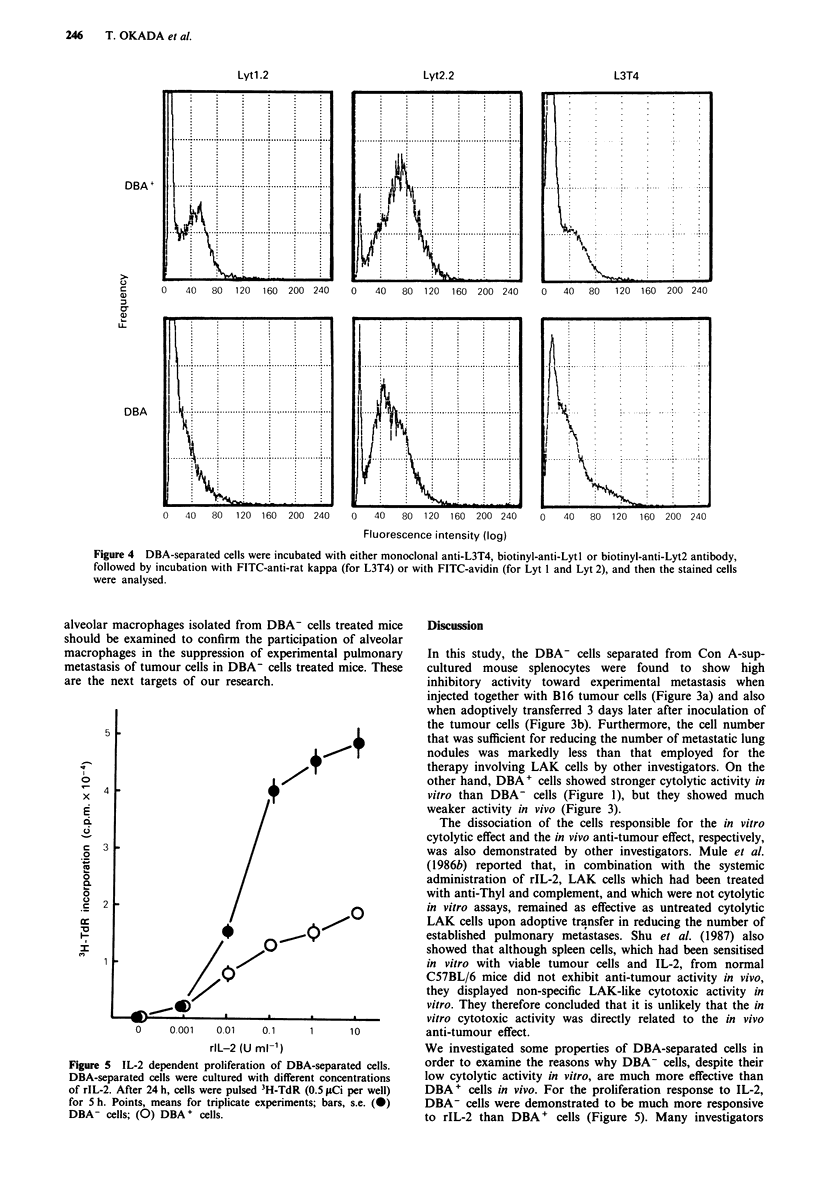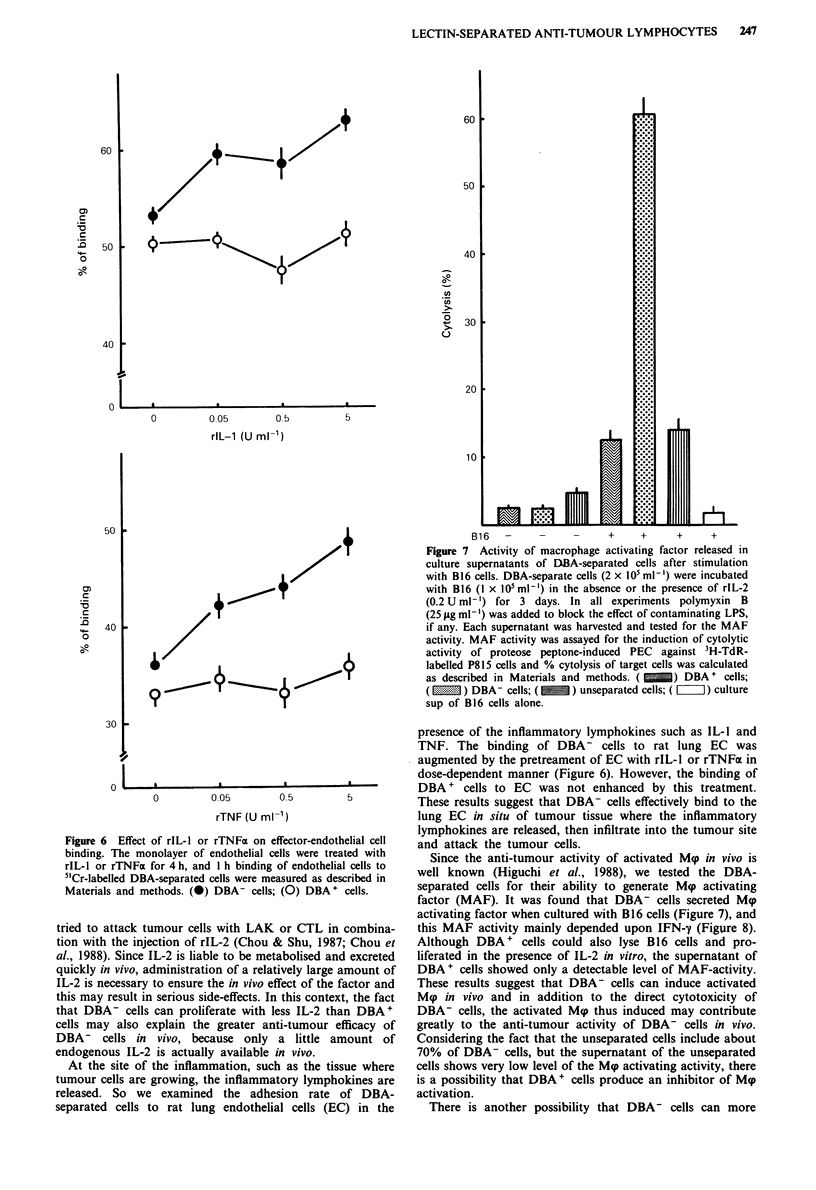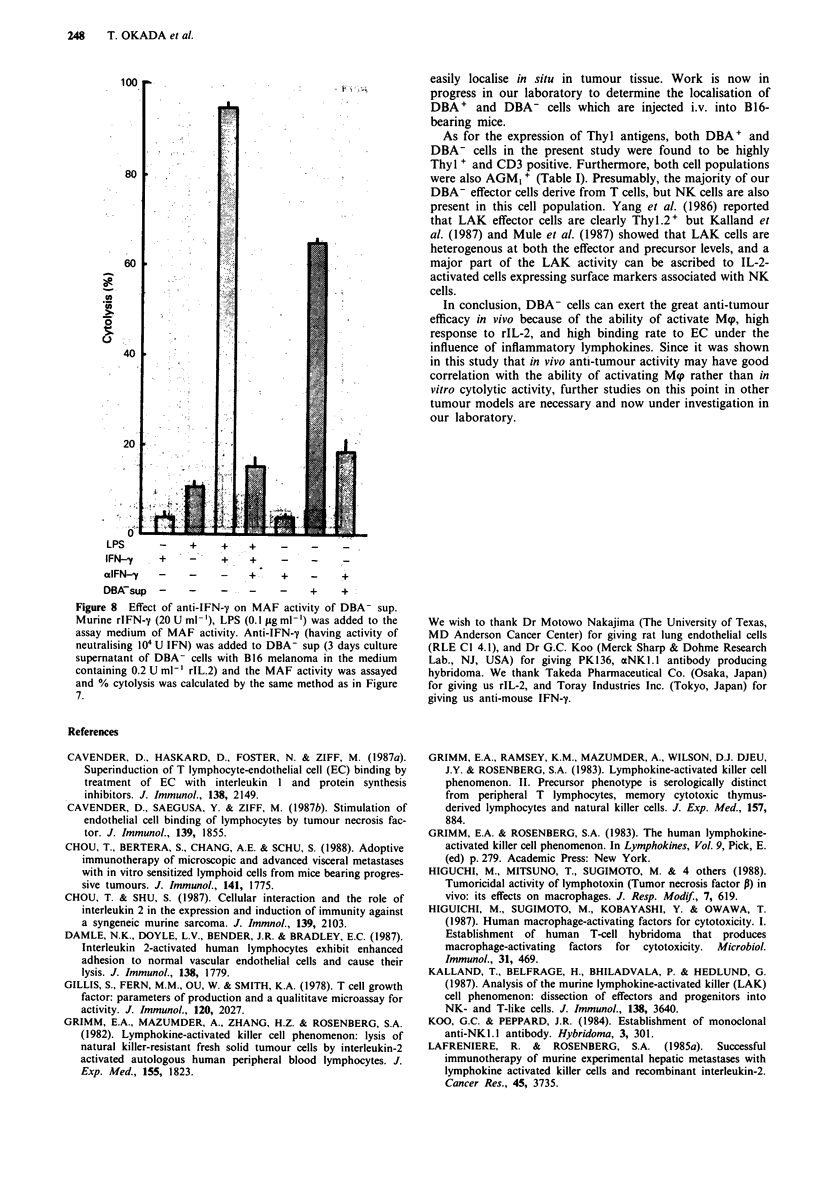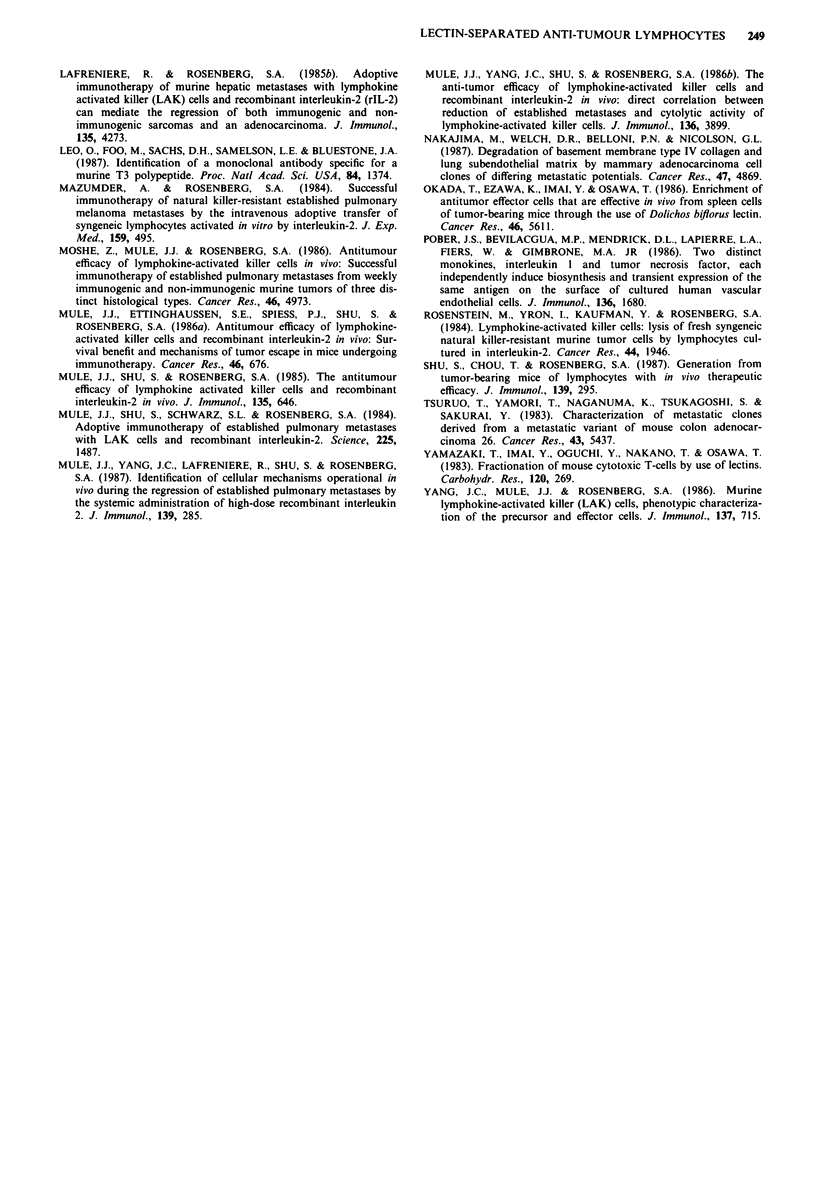# Anti-tumour efficacy of mouse spleen cells separated with Dolichos biflorus lectin (DBA) in experimental pulmonary metastasis of B16 melanoma cells.

**DOI:** 10.1038/bjc.1990.45

**Published:** 1990-02

**Authors:** T. Okada, M. Higuchi, M. Takano, T. Maruyama, Y. Imai, T. Osawa

**Affiliations:** Division of Chemical Toxicology and Immunochemistry, Faculty of Pharmaceutical Sciences, University of Tokyo, Japan.

## Abstract

Anti-tumour effector cells were generated through 4 days culture of normal C57BL/6 splenocytes in a medium containing concanavalin A supernatant and then fractionated with Dolichos biflorus lectin (DBA) into DBA+ (agglutinable with DBA) and DBA- (non-agglutinable with DBA) cells. The DBA- cells, infused intravenously into mice together with B16 melanoma cells, or adoptively transferred into mice 3 days after the injection of B16 cells, caused a marked decrease in the number of lung nodules, while the DBA+ cells exerted no effect. On the other hand, the DBA+ cells exhibited higher cytolytic activity in vitro than the DBA- cells in short-term 51Cr-release assays. Then, we analysed the mechanism of the strong anti-tumour activity of DBA- cells in vivo. We found that DBA- cells showed higher response to recombinant interleukin-2 (rIL-2) than DBA+ cells and proliferated very well with a small amount of IL-2. In addition, DBA- cells adhered more strongly to lung endothelial cells than DBA+ cells in response to rIL-1 or rTNF. Furthermore, DBA- cells produced larger amounts of macrophage activating factor (MAF) including IFN-gamma when cultured with B16 melanoma. Taken together, our results show that DBA- cells are effective in reducing experimental pulmonary metastases not only by the direct lytic activity but also by the indirect killing activity through the activated macrophage.


Br. J. Cancer (1990), 61, 241-249 © Macmillan Press Ltd., 1990

### Anti-tumour efficacy of mouse spleen cells separated with *Dolichos biflorus* lectin (DBA) in experimental pulmonary metastasis of B16 melanoma cells

T. Okada, M. Higuchi, M. Takano, T. Maruyama, Y. Imai & T. Osawa

*Division of Chemical Toxicology and Immunochemistry, Faculty of Pharmaceutical Sciences, University of Tokyo, Bunkyo-ku, Tokyo 113, Japan.*

**Summary** Anti-tumour effector cells were generated through 4 days culture of normal C57BL/6 splenocytes in a medium containing concanavalin A supernatant and then fractionated with *Dolichos biflorus* lectin (DBA) into $DBA^+$ (agglutinable with DBA) and $DBA^-$ (non-agglutinable with DBA) cells. The $DBA^-$ cells, infused intravenously into mice together with B16 melanoma cells, or adoptively transfered into mice 3 days after the injection of B16 cells, caused a marked decrease in the number of lung nodules, while the $DBA^+$ cells exerted no effect. On the other hand, the $DBA^+$ cells exhibited higher cytolytic activity *in vitro* than the $DBA^-$ cells in short-term $^{51}$Cr-release assays. Then, we analysed the mechanism of the strong anti-tumour activity of $DBA^-$ cells *in vivo*. We found that $DBA^-$ cells showed higher reponse to recombinant interleukin-2 (rIL-2) than $DBA^+$ cells and proliferated very well with a small amount of IL-2. In addition, $DBA^-$ cells adhered more strongly to lung endothelial cells than $DBA^+$ cells in response to rIL-1 or rTNF. Furthermore, $DBA^-$ cells produced larger amounts of macrophage activating factor (MAF) including IFN-γ when cultured with B16 melanoma. Taken together, our results show that $DBA^-$ cells are effective in reducing experimental pulmonary metastases not only by the direct lytic activity but also by the indirect killing activity through the activated macrophage.

In previous studies, we fractionated splenocytes of X5563 tumour-bearing mice, which had been treated with the supernatant of mouse splenocytes cultured with concanavalin A (Con A sup), with *Dolichos biflorus* lectin (DBA) into $DBA^+$ (agglutinable with DBA) and $DBA^-$ (non-agglutinable with DBA) cells and demonstrated that $DBA^-$ cells showed strong antitumour activity against X5563 *in vivo* when injected into mice together with human recombinant interleukin-2 (rIL-2), despite the fact that $DBA^+$ cells showed stronger cytolytic activity *in vitro* than $DBA^-$ cells (Okada *et al.*, 1986).

Recent studies have shown that murine splenocytes or human peripheral blood lymphocytes, on incubation *in vitro* in the presence of IL-2, acquire the ability to lyse a variety of fresh syngeneic murine and autologous human tumour cells in short-term $^{51}$Cr-release assays. These lymphokine-activated killer (LAK) cells are distinct from natural killer (NK) cells or classical cytotoxic T-cells, and are lytic toward allogenic tumour target cells from murine and human sources as well (Grimm *et al.*, 1982, 1983; Grimm & Rosenberg, 1983; Rosenstein *et al.*, 1984).

The adoptive transfer of syngeneic LAK cells in conjunction with the systemic administration of rIL-2 reduced the number of pulmonary and hepatic metastases from murine tumours of different histological types (Lafreniere & Rosenberg, 1985*a*, *b*; Mule *et al.*, 1984, 1985, 1986*a*; Moshe *et al.*, 1986).

In the present study, we examined the ability of Con A sup-cultured cells, separated with the use of DBA, to reduce the number of experimental pulmonary metastases in a murine model. Then we investigated the mechanism of anti-tumour activity of $DBA^-$ cells *in vivo*, in terms of the following characteristics of the effector cells: (1) the proliferation rate in response to rIL-2; (2) the lymphokine producing activity; (3) the binding activity to lung endothelial cells under inflammatory conditions.

Correspondence: T. Osawa.
Received 20 March 1989; and in revised form 12 June 1989.

### Materials and methods

### *Mice*

Female C57BL/6, BALB/c and DBA/2 mice (7-12 weeks old) were used in these experiments. The mice were obtained from Charles River Japan Inc. (Kanagawa, Japan).

### *Tumours*

The B16 melanoma tumour line used was of C57BL/6 origin. B16 tumour cells ($1 \times 10^5$) were injected into the tail veins of C57BL/6 mice. After 21-23 days, the animals were killed, their lungs harvested and then the pulmonary nodules counted. Complete enumeration of the experimental metastases was possible because they comprised black nodules on the surface of the lungs. This tumour line was maintained in RPMI-1640 medium supplemented with 10% heat-inactivated fetal calf serum (FCS; MA Bioproducts, Walkersville, MD, USA), 20 mM *N*-2-hydroxyethylpiperazine-*N*′-2-ethanesulphonic acid (HEPES), 2 mM glutamine (Wako Pure Chemical Co., Tokyo, Japan), penicillin (100 $Ul^{-1}$; Sigma Chemical Co., St Louis, MO, USA) and streptomycin (100 $\mu g\ ml^{-1}$; Sigma Chemical Co.). Cultured cells were harvested by treatment with $Ca^{2+}/Mg^{2+}$-free Hank's balanced salt solution containing 0.05% trypsin and 0.02% disodium EDTA at 37°C for 5 min. RPMI-1640 medium containing 10% FCS was added to stop the reaction and then the cells were washed twice with RPMI-1640 medium. Cells were resuspended in the appropriate buffer at the appropriate concentration and used for inoculation.

### *Con A sup*

Spleen cells from female BALB/c mice (8-12 weeks old) were treated with modified Gey's BSS, to lyse erythrocytes, and then cultured at 37°C under 5% $CO_2$ in air for 22-24 h in RPMI-1640 medium supplemented with $5 \times 10^{-5}$ M 2-mercaptoethanol (Sigma), 4 mM glutamine, 1 mM sodium pyruvate, 20 mM HEPES (pH 7.2), 1% non-essential amino acids (GIBCO), penicillin (100 U $ml^{-1}$) and streptomycin

(100 $\mu g\ ml^{-1}$) (complete medium) containing Con A (2.5 $\mu g\ ml^{-1}$) and 2.5% FCS. Then the supernatant was filtered through a membrane filter (pore size, 0.2 $\mu m$; Toyo Roshi, Tokyo, Japan), and stored at $-20°C$ until use. The supernatant (Con A sup) thus prepared was generally used for the cell cultures.

*Assay for IL-2 activity*

The IL-2 activity present in the Con A sup prepared by the method described above was determined in a standard micro-assay method based upon the IL-2 dependent proliferation of CTLL-2 cells (Gillis *et al.*, 1978), which were cultured in flat-bottomed microplate wells in complete medium (200 $\mu l$). Each well contained $10^4$ CTLL cells together with log 2 dilution (ranging from 3% to 50% by volume) of the Con A sup. As a positive control, rIL-2 (Takeda Chemical Industries Ltd, Osaka, Japan) was added to the cultures. The activity of the rIL-2 preparation was defined as 1 U per 27 ng by the supplier. After a 24 h incubation (37°C in a humidified atmosphere of 5% $CO_2$ in air) the microplate wells were pulsed with 0.5 $\mu Ci$ of $^3H$-TdR (15 Ci $mmol^{-1}$; New England Nuclear, Boston, MA, USA). Cultures were harvested 6 h later onto glass fibre filter strips and $^3H$-TdR incorporation was determined with a liquid scintillation counter (LCS-700, Aloka, Tokyo, Japan).

*Generation of cytotoxic effector cells*

The spleens were removed from female C57BL/6 mice and then teased into a single cell suspension in RPMI-1640 medium. The cells were then centrifuged and the pellet resuspended in modified Gey's BSS for 3–4 min at room temperature, to lyse erythrocytes. The cells were then washed three times and cultured at 37°C under 5% $CO_2$ in air in 24-well plates (no. 3424; Costar, Cambridge, MA, USA) at a concentration of $2 \times 10^6$ cells $ml^{-1}$ with 10% Con A sup. On day 3, the culture supernatant was removed, and the cells were harvested, washed and then recultured in the same medium containing 10% Con A sup. Two days later, the cells were harvested and separated with the DBA lectin (EY Laboratory, San Mateo, CA, USA) before i.v. injection or *in vitro* cytotoxicity assays.

*Separation of cytotoxic effector cells through the use of the DBA*

The Con A-sup-cultured cytotoxic effector cells were separated through rosetting with DBA-coupled sheep red blood cells (SRBC) followed by Ficoll-Urografin density gradient centrifugation as previously described (Okada *et al.*, 1986). The $DBA^+$ (rosette-forming cells with DBA-SRBC) and $DBA^-$ (non-rosette-forming cells with DBA-SRBC) cells were treated with modified Gey's BSS, to lyse SRBC, and then washed with a solution of 50 mM *N*-acetyl-D-galactosamine, a haptenic sugar for the DBA lectin.

*$^{51}Cr$-release of cytotoxicity assay*

The 4 h chromium release assay was used in this study. Varying numbers of effector cells (100 $\mu l$) and $10^4$ viable $^{51}Cr$-labelled tumour target cells were plated in the wells of 96-well round bottomed microplated (Sumitomo, Osaka, Japan) in a total volume of 200 $\mu l$. The plates were centrifuged at 100 *g* for 1 min and then incubated at 37°C under 5% $CO_2$ in air for 4 h. Then the plates were centrifuged again at 250 *g* for 5 min, and the supernatants (100 $\mu l$) were harvested and subjected to radioactivity counting with a gamma-counter (ARC-500 auto well gamma system; Aloka, Tokyo, Japan). The spontaneous release was determined by incubating $^{51}Cr$-labelled target cells alone, and the maximum release was determined after lysing the target cells in 0.4 N NaOH. The percentage of specific $^{51}Cr$ release was calculated according to the following formula:

$$\% \text{ of specific } ^{51}Cr \text{ release} = \frac{\text{experimental release (c.p.m.)} - \text{spontaneous release (c.p.m.)}}{\text{maximum release (c.p.m.)} - \text{spontaneous release (c.p.m.)}} \times 100$$

The spontaneous release was less than 10% B16 of the maximum release in all experiments.

*Adoptive immunotherapy models*

C57BL/6 mice were given i.v. injections of $1 \times 10^5$ melanoma cells in 200 $\mu l$ of RPMI-1640 medium. Cytotoxic effector cells were suspended at $4-5 \times 10^6$ cells in 200 $\mu l$ of RPMI-1640 medium and then injected into the tail vein together with the tumour or 3 days after the tumour cell injection. On days 21–23 after the tumour cell injection, the mice were killed for enumeration of metastatic pulmonary nodules. The nodules were counted in a blind fashion, i.e. without knowledge of the treatment. Lungs with metastases too numerous to count upon autopsy were assigned an arbitrary value of 280, since we were able to count reliably only up to about 280 metastases per lung.

*Phenotyping of cytotoxic effector cells*

DBA-separated or unseparated cells ($1 \times 10^6$) were preincubated on ice for 1 h (first incubation) with 200 $\mu l$ of monoclonal biotinyl anti-Lyt1.2 (final dilution, 1/50) or monoclonal biotinyl anti-Lyt2.2 (final dilution, 1/50) (Becton Dickinson, Mountainview, CA, USA) in PBS containing 0.1% $NaN_3$ and 0.1% bovine serum albumin (PBS-$NaN_3$-BSA), and then they were washed twice and treated with 200 $\mu l$ of fluorescein isothiocyanate (FITC)-conjugated avidin (final dilution, 1/100) (Vector Laboratories, Burlingame, CA, USA) on ice for 30 min (second incubation).

When staining cells for analysis of L3T4 antigens, the first incubation was performed with monoclonal anti-L3T4 (final dilution, 1/10; Becton) and the second with FITC-conjugated anti-rat kappa (final dilution, 1/50; Becton).

When staining cells for analysis of the affinity of the DBA lectin, the first incubation was performed with biotinyl DBA (final dilution, 15 $\mu g\ ml^{-1}$; Vector) for 30 min, and the second with FITC-conjugated avidin (final dilution 1/100; Vector Lab.).

In the case of staining cells for analysis of asialo $GM_1$ ($AGM_1$), the first incubation was performed with anti-$AGM_1$ (final dilution, 1/250; Wako Chemical Co., Osaka, Japan) and the second with FITC-goat anti-rabbit Ig (IgA + IgG + IgM) (final dilution, 1/100; Cappel, West Chester, PA, USA).

We examined the expression of T cell receptor on effector cells by staining them with an anti-$T_3$ monoclonal antibody 145-2C11 (Leo *et al.*, 1987) (final dilution, 1/20) for the first incubation, and with FITC-goat anti-hamster IgG (final dilution, 1/100; EY Laboratory, San Mateo, CA, USA) for the second. The expression of NK1.1 on effector cells was examined by staining them with the supernatant of anti-NK1.1-producing PK136 hybridoma (Koo & Peppard, 1984) (final dilution, 1/10) for the first incubation, and with FITC goat anti-mouse IgG (Fc fragment specific, final dilution, 1/50; Cappel) for the second.

Staining of cells for analysis for Thy1.2 was performed by first incubating the cells on ice for 1 h with FITC-conjugated anti-mouse Thy1.2 (final dilution, 1/100; Bio-Yeda, Rehovot, Israel), and then, each lot of cells was analysed using a cell sorter (FCS-1; Japan Spectroscopic Co. Ltd, Tokyo, Japan) after washing three times with PBS-$NaN_3$-BSA.

*Binding to endothelial cells (EC)*

EC monolayer adhesion assay (Damle *et al.*, 1987; Cavender *et al.*, 1987*a*, *b*) was performed as follows. Briefly, to a monolayer of rat lung endothelial cells established by Nakajima *et al.* (1987) in a 96-well flat bottomed plate was added various doses of rIL-1 or rTNFα (200 $\mu l$ per well). After 4 h incubation (37°C, 5% $CO_2$ in air), the plate was washed twice, and $^{51}$Cr-labelled DBA-separated cells ($4 \times 10^5$ per 200 µl per well) were added. After 1 h incubation (37°C, 5% $CO_2$) the plate was washed three times with a warm medium to remove non-adherent cells, and 1% Triton X-100 (200 µl per well) was added. Then the plate was incubated for 10 min at room temperature. The supernatant (100 µl per well) was harvested and assayed for radioactivity with a gamma-counter (ARC-500 auto well gamma system; Aloka, Tokyo, Japan). The percentage of the effector cells which bound to EC was calculated with the following formula:

$$\% \text{ T-EC binding} = \frac{\text{c.p.m. 0.1 ml lysate}}{\text{c.p.m. in original lymphocyte suspension}} \times 100$$

*Macrophage activating factor (MAF) sample*

DBA-separated cells ($2 \times 10^5 \, ml^{-1}$) were cultured with the B16 melanoma ($1 \times 10^5 \, ml^{-1}$) in a 24-well plate (Costar no. 3424, Cambridge, MA, USA) in complete medium including rIL-2 ($0.2 \, Uml^{-1}$). The supernatant was harvested after 3 days, and stored at −20°C until assaying for MAF activity.

*Assay for macrophage activating factor activity for cytotoxicity (MAF-C activity)*

MAF-C activity was assayed essentially by the method of Higuchi *et al.* (1987). Briefly, peritoneal exudate cells (PEC) were obtained from DBA/2 mice 3 days after intraperitoneal injection of proteose peptone. These PEC ($1 \times 10^5$ per 100 µl per well) were plated in a 96-well microplate (Sumitomo, Osaka, Japan) in RPMI-1640 medium including 10% FCS. After 2 h incubation (37°C, 5% $CO_2$ in air), the supernatant was removed, and the MAF sample was added. When the effect of anti-IFN-γ was examined, anti-IFN-γ (1/100 rabbit anti-mouse IFN-γ having activity of neutralising $10^6$ U IFN $ml^{-1}$; Toray Industries Inc., Tokyo, Japan) was added to the sample. The plate was then incubated for 24 h (37°C, 5% $CO_2$ in air) before the addition of $^3$H-TdR prelabelled P815 cells ($10^4$ cells per 200 µl per well). After 48 h incubation (37°C, 5% $CO_2$ in air), the supernatant (100 µl per well) was harvested and $^3$H-TdR release was determined with a scintillation counter. Control release was determined by incubating $^3$H-TdR-labelled target cells alone, and the total count was determined with target cells dissolved with 1% sodium dodecyl sulphate. The percentage of MAF-C activity was determined with the following formula.

$$\text{MAF-C activity (\%)} = \frac{\text{release in test sample} - \text{release in control sample}}{\text{total count} - \text{release in control sample}} \times 100$$

### Results

*In vitro cytolytic activity of DBA-separated effector cells*

Since we previously demonstrated that alloreactive CTL (Yamazaki *et al.*, 1983) and Con A sup-cultured splenocytes from X5563 tumour-bearing C3H/HeN mice (Okada *et al.*, 1986) could be enriched in the cell fraction having affinity for DBA (DBA$^+$ cells), when examined *in vitro* against X5563 cells in a 4 h $^{51}$Cr release assay, we investigated whether or not Con A sup-cultured splenocytes from normal mice were enriched in the DBA$^+$ fraction in the present study.

To induce cytotoxic effector cells, we used normal C57BL/6 mouse spleen cells expanded *in vitro* in a Con A sup-containing medium. The IL-2 activity present in 10% (v/v) Con A sup was about $0.2 \, U \, ml^{-1}$ when determined in a standard microassay using the IL-2 dependent CTL line, CTLL-2. Splenocytes were cultured for 4 days in the presence of 10% Con A sup (v/v) and then separated into DBA$^+$ and DBA$^-$ fractions by means of rosette formation using DBA coupled SRBC. The distribution into the DBA$^+$ and DBA$^-$ fractions was 29.6±2.4% and 70.4±2.4%, respectively (mean ± s.e. for seven experiments). To confirm that DBA$^+$ cells were highly enriched in the DBA$^+$ fraction, separated and unseparated cells were incubated with the biotinyl-DBA followed by incubation with FITC-avidin, and then the stained cells ($10^4$ cells per sample) were analysed by flow cytometry. As shown in Table I, DBA$^+$ and DBA$^-$ cells were highly enriched in the DBA$^+$ and DBA$^-$ fractions, respectively.

The cytolytic activity of these cells was determined by the standard 4-h $^{51}$Cr-release assay. As shown in Figure 1a, the DBA$^+$ cells showed stronger cytolytic activity against the B16 melanoma than the DBA$^-$ cells *in vitro* (E/T = 25; Student's *t* test, $P<0.01$). We also assessed the cytolytic activity of Con A sup-cultured splenocytes from B16 tumour-bearing C57BL/6 mice against B16 melanoma cells *in vitro*. Viable B16 tumour cells ($1 \times 10^6$ per mouse) were inoculated subcutaneously into the backs of C57BL/6 mice. Seven days later, splenocytes were prepared from these mice and cultured for 4 days in the same manner. They were separated with the DBA and then separated cells were tested for lytic activity against B16 cells. As shown in Figure 1b the DBA$^+$ cells also exhibited stronger cytolytic activity than the DBA$^-$ cells in this case (E/T = 25; Student's *t* test, $P<0.01$).

We then examined *in vitro* cytolytic activity of Con A sup-cultured splenocytes from normal mice against NK sensitive target cells, YAC-1 and NK resistant target cells, P815 and EL-4. As shown in Figure 2, the DBA$^+$ cells also showed significantly stronger cytolytic activity than the DBA$^-$ cells in these cases (E/T = 15; Student's *t* test, $P<0.001$).

*In vivo anti-tumour activity of DBA-separated cells*

To evaluate the anti-tumour efficacy of the DBA-separated cells *in vivo*, DBA-separated cells ($4 \times 10^6$) were intravenously injected into C57BL/6 mice together with B16 cells ($1 \times 10^5$) injection (Winn assay). Figure 3a shows the number of experimental pulmonary metastases determined for each group, in a blind fashion. The infusion of DBA$^-$ cells caused a marked decrease in the number of lung metastases detectable on day 21, compared with that in mice given DBA$^+$ cells (Student's *t* test, $P<0.01$), or unseparated cells (Student's *t* test, $P<0.01$) or without treatment (Student's *t* test, $P<0.001$). Since DBA$^-$ cells showed significantly stronger anti-tumour effect than DBA$^+$ cells in the Winn assays, then we investigated the effect of adoptively transferred DBA-separated cells on B16 tumour nodules in lungs that had already undergone micro-metastasis. B16 cells ($1 \times 10^5$) were intravenously injected into mice, and 3 days later, DBA-separated cells ($5 \times 10^6$) were i.v. transferred. On day 23 after the tumour cells injection, the number of experimental pulmonary metastases was determined. Figure 3b shows that the adoptive immunotherapy for established 3-day pulmonary B16 metastases with DBA$^-$ cells resulted in a significant reduction in the number of pulmonary metastatic nodules compared with the transfer of unseparated or DBA$^+$ cells (Student's *t* test, $P<0.001$). Entirely similar results to those in Figure 3b were obtained in three independent experiments.

It was demonstrated by Mazumder and Rosenberg (1984) that injection of LAK cells ($1 \times 10^8$) could significantly decrease the number of experimental pulmonary B16 melanoma metastases in mice injected with $2 \times 10^5$ B16 cells

**Table I** Surface phenotypes of DBA-separated cells[a]

| *Cells* | *DBA* | *Thy1.2* | *2C11* | *L3T4* | *Lyt1.2* | *Lyt2.2* | *AGM$_1$* | *NK1.1* |
|---|---|---|---|---|---|---|---|---|
| DBA$^+$ | 90.4 | 98.3 | 63.6 | 40.4 | 41.5 | 92.6 | 79.1 | 9.1 |
| DBA$^-$ | 11.5 | 96.6 | 72.5 | 66.0 | 36.3 | 86.3 | 90.4 | 12.2 |
| Unseparated | 27.2 | 96.1 | 67.8 | n.t. | 40.6 | 91.7 | n.t.[b] | 10.8 |

Values are % positive cells. [a]DBA-separated cells were analysed for the expression of surface markers by the flow cytometry. [b]n.t., not tested.

**Figure 1** Cytolytic activity of DBA-separated cells against B16 melanoma cells. Spleen cells from normal C57BL/6 mice were separated with the DBA lectin after culturing for 4 days in the presence of 10% (v/v) Con A sup. **a**, The cytolytic activity against B16 cells of DBA$^+$ (●), DBA$^-$ (○) and unseparated (□) cells was examined by means of the 4 h $^{51}$Cr-release assay. Points, means for triplicate experiments; bars, s.e. **b**, Cytolytic activity of DBA-separated spleen cells from B16-bearing mice cultured with Con A sup. B16 cells ($10^6$) were subcutaneously inoculated into the backs of C57BL/6 mice, and 7 days later, the spleen cells were cultured for 4 days in the presence of 10% (v/v) Con A sup. These cells were separated into DBA$^+$ (●), DBA$^-$ (○) and unseparated (□) cells, and then examined by means of the 4 h $^{51}$Cr-release assay. Points, means for triplicate experiments; bars, s.e.

**Figure 2** Cytolytic activity of DBA-separated cells against B16 melanoma (**a**), YAC-1 cells (**b**), P815 cells (**c**) and EL-4 cells (**d**). The effector cells are the same as Figure 1a. The cytolytic activity against each target of DBA$^+$ (●), DBA$^-$ (○) and unseparated (□) cells was examined by the 4 h $^{51}$Cr-release assay. Points, means for triplicate experiments; bars, s.e.

3 days before the injection of LAK cells, but in the present study, a much lower number of DBA$^-$ effector cells was found to be sufficient to decrease the number of lung metastases. On the other hand, DBA$^+$ or unseparated cells were ineffective on adoptive transfer under our experimental conditions.

We obtained the same results for another mouse experimental pulmonary metastasis model; DBA-separated BALB/c effector cells against NL-17 cells (M. Takano, T. Okada and T. Osawa, unpublished results) which are a highly metastatic cell line established from colon adenocarcinoma 26 cells (Tsuruo *et al.*, 1983).

### *Phenotypes of DBA-separated cells*

We then determined the surface phenotypes of the DBA-separated cells. As shown in Table I, most of the DBA$^+$, DBA$^-$ and unseparated cells were highly Thy1.2$^+$. Although both separated populations (DBA$^+$ and DBA$^-$) were found to express the L3T4, Lyt1.2 and Lyt 2.2 antigens (Table I and Figure 4), the DBA$^-$ fraction contained more L3T4 positive cells than the DBA$^+$ fraction, and the DBA$^+$ fraction consisted of the cells which are a little more positive in Lyt 1 and Lyt 2 antigens than the DBA$^-$ fraction. Both DBA$^+$ and DBA$^-$ cells were AGM$_1$$^+$, and these cell populations contained NK1.1$^+$ cells. As for the T cell receptor, both DBA$^+$ and DBA$^-$ cells were 2C11$^+$.

### *Growth rate of DBA-separated cells in the presence of IL-2*

To examine the reason why DBA$^-$ cells, despite their low cytolytic activity *in vitro*, are markedly effective *in vivo*, we

**Figure 3** **a**, DBA-separated cells ($4 \times 10^6$) were injected i.v. into mice together with B16 cells ($1 \times 10^5$). After 21 days, the mice were killed and then the number of lung nodules was determined. **b**, DBA-separated cells ($5 \times 10^6$) were injected i.v. into mice that had been injected 3 days previously with B16 cells ($1 \times 10^5$). Twenty-three days after the tumour cell injection, the number of lung metastases was determined.

first examined the response of separated cells to rIL-2. As shown in Figure 5, $DBA^-$ cells were more responsive to rIL-2 than $DBA^+$ cells. These results were quite reproducible in several independent experiments. Since there seems to be very little IL-2 *in vivo*, the high responsiveness of $DBA^-$ cells to IL-2 even in very low concentrations of the factor may be very advantageous for the cells to proliferate *in vivo*.

*Binding to endothelial cells of blood vessel under inflammatory condition*

Infiltration of leukocytes into inflammation sites, such as tumour metastasis sites, is related to their ability to bind to endothelial cells of blood vessels under inflammatory condition. The binding is well known to be regulated by cytokines such as IL-1 and IFN (Cavender *et al.*, 1987*a*, *b*). *In vitro* study showed that the pretreatment of endothelial cells with rIL-1 or rTNF augments their adhesion to lymphocytes or IL-2 cultured lymphocytes (Damle *et al.*, 1987; Cavender *et al.*, 1987*a*, *b*), and the lymphocyte-binding reached a plateau at the concentration of 1 U $ml^{-1}$ of IL-1 (Pober *et al.*, 1986) or TNF (Cavender *et al.*, 1987*b*). We tested the rate of the adhesion of the DBA-separated cells to the endothelial cells of blood vessels in rat lung after treatment of the endothelial cells with various doses (0–5 U $ml^{-1}$) of rIL-1 or rTNFα. Since mouse lung endothelial cells were not available, rat lung endothelial cells kindly donated by Dr M. Nakajima (MD Anderson Hospital, Houston, TX, USA) were used in this study. As shown in Figure 6, rIL-1 or rTNFα increased the adhesion of $DBA^-$ cells to endothelial cells while these lymphokines had no effect on the adhesion of $DBA^+$ cells to endothelial cells. At the site of the tumour metastases, the inflammatory lymphokines such as IL-1 or TNF may be secreted. These results indicate that $DBA^-$ cells have an ability to attach to endothelial cells under the influence of IL-1 or TNF, and they can infiltrate advantegeously into the tumour metastasis sites. However, similar studies on mouse lung endothelial cells are still necessary, because there is a possibilty that cell surface adhesion molecules are different between mouse and rate endothelial cells.

*Indirect anti-tumour effect through the elaboration of lymphokines*

Furthermore, since there is a possibilty that $DBA^-$ cells act indirectly through activation of other types of cells by secretion of certain lymphokines, experiments were performed to find out whether DBA-separated cells secrete the macrophage activating factor (MAF). DBA-separated or unseparated cells were cultured with the B16 melanoma in the presence of rIL-2 for several days. Then the supernatants were tested for MAF activity. MAF activity was estimated by the induction of cytolytic activity of proteose peptone-induced macrophages (Mφ) against P815 cells. The culture medium used in these experiments contained polymyxin B to block the effect of contaminating LPS, if any. As shown in Figure 7, $DBA^-$ cells produced a large amount of MAF when cultured with the B16 melanoma in the presence of rIL-2 for 3 days. On the other hand, $DBA^+$ or unseparated cells produced only a detectable level of MAF. In the absence of the B16 melanoma, neither $DBA^+$ nor $DBA^-$ cells released MAF, and no MAF was produced from B16 cells alone under these cultures conditions. If rIL-2 was not added to the culture medium, all the effector cells died in 2 days and no MAF was secreted. These results suggested that $DBA^-$ cells were stimulated by the B16 melanoma in the presence of rIL-2 and secreted MAF in the culture medium. The production of MAF was detectable on day 2 and reached a peak on day 3. After 4 or 5 days, the viability of effector cells decreased and no MAF activity was detected.

We then examined characteristics of the MAF activity in $DBA^-$ supernatant. As shown in Figure 8, neither LPS nor murine rIFN-γ showed MAF activity by themselves, but showed MAF activity synergistically. This effect was blocked by anti-IFN-γ antibody. When anti-IFN-γ was added to $DBA^-$ sup, MAF activity was almost completely diminished. These results show that MAF activity in $DBA^-$ sup is mainly mediated by IFN-γ, and suggest the possibility that $DBA^-$ cells secrete IFN-γ *in vivo* and the activated Mφ thus induced may also contribute to the anti-tumour activity. However, direct determinations of IFN-γ in the supernatants of DBA-separated and unseparated cell populations are necessary to verify this assumption. Furthermore, cytotoxic activity of

**Figure 4** DBA-separated cells were incubated with either monoclonal anti-L3T4, biotinyl-anti-Lyt1 or biotinyl-anti-Lyt2 antibody, followed by incubation with FITC-anti-rat kappa (for L3T4) or with FITC-avidin (for Lyt 1 and Lyt 2), and then the stained cells were analysed.

alveolar macrophages isolated from DBA$^-$ cells treated mice should be examined to confirm the participation of alveolar macrophages in the suppression of experimental pulmonary metastasis of tumour cells in DBA$^-$ cells treated mice. These are the next targets of our research.

**Figure 5** IL-2 dependent proliferation of DBA-separated cells. DBA-separated cells were cultured with different concentrations of rIL-2. After 24 h, cells were pulsed $^3$H-TdR (0.5 $\mu$Ci per well) for 5 h. Points, means for triplicate experiments; bars, s.e. (●) DBA$^-$ cells; (○) DBA$^+$ cells.

### Discussion

In this study, the DBA$^-$ cells separated from Con A-sup-cultured mouse splenocytes were found to show high inhibitory activity toward experimental metastasis when injected together with B16 tumour cells (Figure 3a) and also when adoptively transferred 3 days later after inoculation of the tumour cells (Figure 3b). Furthermore, the cell number that was sufficient for reducing the number of metastatic lung nodules was markedly less than that employed for the therapy involving LAK cells by other investigators. On the other hand, DBA$^+$ cells showed stronger cytolytic activity *in vitro* than DBA$^-$ cells (Figure 1), but they showed much weaker activity *in vivo* (Figure 3).

The dissociation of the cells responsible for the *in vitro* cytolytic effect and the *in vivo* anti-tumour effect, respectively, was also demonstrated by other investigators. Mule *et al.* (1986*b*) reported that, in combination with the systemic administration of rIL-2, LAK cells which had been treated with anti-Thyl and complement, and which were not cytolytic *in vitro* assays, remained as effective as untreated cytolytic LAK cells upon adoptive transfer in reducing the number of established pulmonary metastases. Shu *et al.* (1987) also showed that although spleen cells, which had been sensitised *in vitro* with viable tumour cells and IL-2, from normal C57BL/6 mice did not exhibit anti-tumour activity *in vivo*, they displayed non-specific LAK-like cytotoxic activity *in vitro*. They therefore concluded that it is unlikely that the *in vitro* cytotoxic activity was directly related to the *in vivo* anti-tumour effect.

We investigated some properties of DBA-separated cells in order to examine the reasons why DBA$^-$ cells, despite their low cytolytic activity *in vitro*, are much more effective than DBA$^+$ cells *in vivo*. For the proliferation response to IL-2, DBA$^-$ cells were demonstrated to be much more responsive to rIL-2 than DBA$^+$ cells (Figure 5). Many investigators

**Figure 6** Effect of rIL-1 or rTNFα on effector-endothelial cell binding. The monolayer of endothelial cells were treated with rIL-1 or rTNFα for 4 h, and 1 h binding of endothelial cells to $^{51}$Cr-labelled DBA-separated cells were measured as described in Materials and methods. (●) DBA$^-$ cells; (O) DBA$^+$ cells.

**Figure 7** Activity of macrophage activating factor released in culture supernatants of DBA-separated cells after stimulation with B16 cells. DBA-separate cells (2 × 10$^5$ ml$^{-1}$) were incubated with B16 (1 × 10$^5$ ml$^{-1}$) in the absence or the presence of rIL-2 (0.2 U ml$^{-1}$) for 3 days. In all experiments polymyxin B (25 μg ml$^{-1}$) was added to block the effect of contaminating LPS, if any. Each supernatant was harvested and tested for the MAF activity. MAF activity was assayed for the induction of cytolytic activity of proteose peptone-induced PEC against $^3$H-TdR-labelled P815 cells and % cytolysis of target cells was calculated as described in Materials and methods. ( ) DBA$^+$ cells; ( ) DBA$^-$ cells; ( ) unseparated cells; ( ) culture sup of B16 cells alone.

tried to attack tumour cells with LAK or CTL in combination with the injection of rIL-2 (Chou & Shu, 1987; Chou *et al.*, 1988). Since IL-2 is liable to be metabolised and excreted quickly *in vivo*, administration of a relatively large amount of IL-2 is necessary to ensure the *in vivo* effect of the factor and this may result in serious side-effects. In this context, the fact that DBA$^-$ cells can proliferate with less IL-2 than DBA$^+$ cells may also explain the greater anti-tumour efficacy of DBA$^-$ cells *in vivo*, because only a little amount of endogenous IL-2 is actually available *in vivo*.

At the site of the inflammation, such as the tissue where tumour cells are growing, the inflammatory lymphokines are released. So we examined the adhesion rate of DBA-separated cells to rat lung endothelial cells (EC) in the presence of the inflammatory lymphokines such as IL-1 and TNF. The binding of DBA$^-$ cells to rat lung EC was augmented by the pretreament of EC with rIL-1 or rTNFα in dose-dependent manner (Figure 6). However, the binding of DBA$^+$ cells to EC was not enhanced by this treatment. These results suggest that DBA$^-$ cells effectively bind to the lung EC *in situ* of tumour tissue where the inflammatory lymphokines are released, then infiltrate into the tumour site and attack the tumour cells.

Since the anti-tumour activity of activated Mφ *in vivo* is well known (Higuchi *et al.*, 1988), we tested the DBA-separated cells for their ability to generate Mφ activating factor (MAF). It was found that DBA$^-$ cells secreted Mφ activating factor when cultured with B16 cells (Figure 7), and this MAF activity mainly depended upon IFN-γ (Figure 8). Although DBA$^+$ cells could also lyse B16 cells and proliferated in the presence of IL-2 *in vitro*, the supernatant of DBA$^+$ cells showed only a detectable level of MAF-activity. These results suggest that DBA$^-$ cells can induce activated Mφ *in vivo* and in addition to the direct cytotoxicity of DBA$^-$ cells, the activated Mφ thus induced may contribute greatly to the anti-tumour activity of DBA$^-$ cells *in vivo*. Considering the fact that the unseparated cells include about 70% of DBA$^-$ cells, but the supernatant of the unseparated cells shows very low level of the Mφ activating activity, there is a possibility that DBA$^+$ cells produce an inhibitor of Mφ activation.

There is another possibility that DBA$^-$ cells can more

**Figure 8** Effect of anti-IFN-γ on MAF activity of DBA⁻ sup. Murine rIFN-γ (20 U $ml^{-1}$), LPS (0.1 μg $ml^{-1}$) was added to the assay medium of MAF activity. Anti-IFN-γ (having activity of neutralising $10^4$ U IFN) was added to DBA⁻ sup (3 days culture supernatant of DBA⁻ cells with B16 melanoma in the medium containing 0.2 U $ml^{-1}$ rIL.2) and the MAF activity was assayed and % cytolysis was calculated by the same method as in Figure 7.

easily localise *in situ* in tumour tissue. Work is now in progress in our laboratory to determine the localisation of $DBA^+$ and $DBA^-$ cells which are injected i.v. into B16-bearing mice.

As for the expression of Thy1 antigens, both $DBA^+$ and $DBA^-$ cells in the present study were found to be highly $Thy1^+$ and CD3 positive. Furthermore, both cell populations were also $AGM_1{}^+$ (Table I). Presumably, the majority of our $DBA^-$ effector cells derive from T cells, but NK cells are also present in this cell population. Yang *et al.* (1986) reported that LAK effector cells are clearly $Thy1.2^+$ but Kalland *et al.* (1987) and Mule *et al.* (1987) showed that LAK cells are heterogenous at both the effector and precursor levels, and a major part of the LAK activity can be ascribed to IL-2-activated cells expressing surface markers associated with NK cells.

In conclusion, $DBA^-$ cells can exert the great anti-tumour efficacy *in vivo* because of the ability of activate Mφ, high response to rIL-2, and high binding rate to EC under the influence of inflammatory lymphokines. Since it was shown in this study that *in vivo* anti-tumour activity may have good correlation with the ability of activating Mφ rather than *in vitro* cytolytic activity, further studies on this point in other tumour models are necessary and now under investigation in our laboratory.

We wish to thank Dr Motowo Nakajima (The University of Texas, MD Anderson Cancer Center) for giving rat lung endothelial cells (RLE Cl 4.1), and Dr G.C. Koo (Merck Sharp & Dohme Research Lab., NJ, USA) for giving PK136, αNK1.1 antibody producing hybridoma. We thank Takeda Pharmaceutical Co. (Osaka, Japan) for giving us rIL-2, and Toray Industries Inc. (Tokyo, Japan) for giving us anti-mouse IFN-γ.

**References**

CAVENDER, D., HASKARD, D., FOSTER, N. & ZIFF, M. (1987*a*). Superinduction of T lymphocyte-endothelial cell (EC) binding by treatment of EC with interleukin 1 and protein synthesis inhibitors. *J. Immunol.*, **138,** 2149.

CAVENDER, D., SAEGUSA, Y. & ZIFF, M. (1987*b*). Stimulation of endothelial cell binding of lymphocytes by tumour necrosis factor. *J. Immunol.*, **139,** 1855.

CHOU, T., BERTERA, S., CHANG, A.E. & SCHU, S. (1988). Adoptive immunotherapy of microscopic and advanced visceral metastases with in vitro sensitized lymphoid cells from mice bearing progressive tumours. *J. Immunol.*, **141,** 1775.

CHOU, T. & SHU, S. (1987). Cellular interaction and the role of interleukin 2 in the expression and induction of immunity against a syngeneic murine sarcoma. *J. Immnol.*, **139,** 2103.

DAMLE, N.K., DOYLE, L.V., BENDER, J.R. & BRADLEY, E.C. (1987). Interleukin 2-activated human lymphocytes exhibit enhanced adhesion to normal vascular endothelial cells and cause their lysis. *J. Immunol.*, **138,** 1779.

GILLIS, S., FERN, M.M., OU, W. & SMITH, K.A. (1978). T cell growth factor: parameters of production and a qualititave microassay for activity. *J. Immunol.*, **120,** 2027.

GRIMM, E.A., MAZUMDER, A., ZHANG, H.Z. & ROSENBERG, S.A. (1982). Lymphokine-activated killer cell phenomenon: lysis of natural killer-resistant fresh solid tumour cells by interleukin-2 activated autologous human peripheral blood lymphocytes. *J. Exp. Med.*, **155,** 1823.

GRIMM, E.A., RAMSEY, K.M., MAZUMDER, A., WILSON, D.J. DJEU, J.Y. & ROSENBERG, S.A. (1983). Lymphokine-activated killer cell phenomenon. II. Precursor phenotype is serologically distinct from peripheral T lymphocytes, memory cytotoxic thymus-derived lymphocytes and natural killer cells. *J. Exp. Med.*, **157,** 884.

GRIMM, E.A. & ROSENBERG, S.A. (1983). The human lymphokine-activated killer cell phenomenon. In *Lymphokines*, *Vol. 9*, Pick, E. (ed) p. 279. Academic Press: New York.

HIGUCHI, M., MITSUNO, T., SUGIMOTO, M. & 4 others (1988). Tumoricidal activity of lymphotoxin (Tumor necrosis factor β) in vivo: its effects on macrophages. *J. Resp. Modif.*, **7,** 619.

HIGUICHI, M., SUGIMOTO, M., KOBAYASHI, Y. & OWAWA, T. (1987). Human macrophage-activating factors for cytotoxicity. I. Establishment of human T-cell hybridoma that produces macrophage-activating factors for cytotoxicity. *Microbiol. Immunol.*, **31,** 469.

KALLAND, T., BELFRAGE, H., BHILADVALA, P. & HEDLUND, G. (1987). Analysis of the murine lymphokine-activated killer (LAK) cell phenomenon: dissection of effectors and progenitors into NK- and T-like cells. *J. Immunol.*, **138,** 3640.

KOO, G.C. & PEPPARD, J.R. (1984). Establishment of monoclonal anti-NK1.1 antibody. *Hybridoma*, **3,** 301.

LAFRENIERE, R. & ROSENBERG, S.A. (1985*a*). Successful immunotherapy of murine experimental hepatic metastases with lymphokine activated killer cells and recombinant interleukin-2. *Cancer Res.*, **45,** 3735.

LAFRENIERE, R. & ROSENBERG, S.A. (1985*b*). Adoptive immunotherapy of murine hepatic metastases with lymphokine activated killer (LAK) cells and recombinant interleukin-2 (rIL-2) can mediate the regression of both immunogenic and non-immunogenic sarcomas and an adenocarcinoma. *J. Immunol.*, **135**, 4273.

LEO, O., FOO, M., SACHS, D.H., SAMELSON, L.E. & BLUESTONE, J.A. (1987). Identification of a monoclonal antibody specific for a murine T3 polypeptide. *Proc. Natl Acad. Sci. USA*, **84**, 1374.

MAZUMDER, A. & ROSENBERG, S.A. (1984). Successful immunotherapy of natural killer-resistant established pulmonary melanoma metastases by the intravenous adoptive transfer of syngeneic lymphocytes activated *in vitro* by interleukin-2. *J. Exp. Med.*, **159**, 495.

MOSHE, Z., MULE, J.J. & ROSENBERG, S.A. (1986). Antitumour efficacy of lymphokine-activated killer cells *in vivo*: Successful immunotherapy of established pulmonary metastases from weekly immunogenic and non-immunogenic murine tumors of three distinct histological types. *Cancer Res.*, **46**, 4973.

MULE, J.J., ETTINGHAUSSEN, S.E., SPIESS, P.J., SHU, S. & ROSENBERG, S.A. (1986*a*). Antitumour efficacy of lymphokine-activated killer cells and recombinant interleukin-2 *in vivo*: Survival benefit and mechanisms of tumor escape in mice undergoing immunotherapy. *Cancer Res.*, **46**, 676.

MULE, J.J., SHU, S. & ROSENBERG, S.A. (1985). The antitumour efficacy of lymphokine activated killer cells and recombinant interleukin-2 *in vivo*. *J. Immunol.*, **135**, 646.

MULE, J.J., SHU, S., SCHWARZ, S.L. & ROSENBERG, S.A. (1984). Adoptive immunotherapy of established pulmonary metastases with LAK cells and recombinant interleukin-2. *Science*, **225**, 1487.

MULE, J.J., YANG, J.C., LAFRENIERE, R., SHU, S. & ROSENBERG, S.A. (1987). Identification of cellular mechanisms operational *in vivo* during the regression of established pulmonary metastases by the systemic administration of high-dose recombinant interleukin 2. *J. Immunol.*, **139**, 285.

MULE, J.J., YANG, J.C., SHU, S. & ROSENBERG, S.A. (1986*b*). The anti-tumor efficacy of lymphokine-activated killer cells and recombinant interleukin-2 *in vivo*: direct correlation between reduction of established metastases and cytolytic activity of lymphokine-activated killer cells. *J. Immunol.*, **136**, 3899.

NAKAJIMA, M., WELCH, D.R., BELLONI, P.N. & NICOLSON, G.L. (1987). Degradation of basement membrane type IV collagen and lung subendothelial matrix by mammary adenocarcinoma cell clones of differing metastatic potentials. *Cancer Res.*, **47**, 4869.

OKADA, T., EZAWA, K., IMAI, Y. & OSAWA, T. (1986). Enrichment of antitumor effector cells that are effective *in vivo* from spleen cells of tumor-bearing mice through the use of *Dolichos biflorus* lectin. *Cancer Res.*, **46**, 5611.

POBER, J.S., BEVILACGUA, M.P., MENDRICK, D.L., LAPIERRE, L.A., FIERS, W. & GIMBRONE, M.A. JR (1986). Two distinct monokines, interleukin 1 and tumor necrosis factor, each independently induce biosynthesis and transient expression of the same antigen on the surface of cultured human vascular endothelial cells. *J. Immunol.*, **136**, 1680.

ROSENSTEIN, M., YRON, I., KAUFMAN, Y. & ROSENBERG, S.A. (1984). Lymphokine-activated killer cells: lysis of fresh syngeneic natural killer-resistant murine tumor cells by lymphocytes cultured in interleukin-2. *Cancer Res.*, **44**, 1946.

SHU, S., CHOU, T. & ROSENBERG, S.A. (1987). Generation from tumor-bearing mice of lymphocytes with *in vivo* therapeutic efficacy. *J. Immunol.*, **139**, 295.

TSURUO, T., YAMORI, T., NAGANUMA, K., TSUKAGOSHI, S. & SAKURAI, Y. (1983). Characterization of metastatic clones derived from a metastatic variant of mouse colon adenocarcinoma 26. *Cancer Res.*, **43**, 5437.

YAMAZAKI, T., IMAI, Y., OGUCHI, Y., NAKANO, T. & OSAWA, T. (1983). Fractionation of mouse cytotoxic T-cells by use of lectins. *Carbohydr. Res.*, **120**, 269.

YANG, J.C., MULE, J.J. & ROSENBERG, S.A. (1986). Murine lymphokine-activated killer (LAK) cells, phenotypic characterization of the precursor and effector cells. *J. Immunol.*, **137**, 715.